# Endoscope-assisted resection of brainstem cavernous malformations

**DOI:** 10.1007/s10143-022-01793-5

**Published:** 2022-05-02

**Authors:** Joachim Oertel, Gerrit Fischer, Stefan Linsler, Matthias Huelser, Christoph Sippl, Fritz Teping

**Affiliations:** Department of Neurosurgery, Saarland University Medical Centre, Kirrbergerstraße, Gebäude 90.5, 66421 Homburg, Saarland Germany

**Keywords:** Neuroendoscopy, Endoscopic neurosurgery, Brainstem, Cavernoma, Cavernous malformation

## Abstract

Targeted surgical precision and minimally invasive techniques are of utmost importance for resectioning cavernous malformations involving the brainstem region. Minimisation of the surgical corridor is desirable but should not compromise the extent of resection. This study provides detailed information on the role of endoscopy in this challenging surgical task. A retrospective analysis of medical documentation, radiologic studies and detailed intraoperative video documentation was performed for all consecutive patients who underwent surgical resection of brainstem cavernous malformations between 2010 and 2020 at the authors’ institution. A case-based volumetry of the corticotomy was performed and compared to cavernoma dimensions. A total of 20 procedures have been performed in 19 patients. Neuroendoscopy was implemented in all cases. The mean size of the lesion was 5.4 (± 5) mm^3^. The average size of the brainstem corticotomy was 4.5 × 3.7 (± 1.0 × 1.1) mm, with a median relation to the cavernoma’s dimension of 9.99% (1.2–31.39%). Endoscopic 360° inspection of the resection cavity was feasible in all cases. There were no endoscopy-related complications. Mean follow-up was 27.8 (12–89) months. Gross-total resection was achieved in all but one case (95%). Sixteen procedures (80%) resulted in an improved or stable medical condition. Eleven patients (61.1%) showed further improvement 12 months after the initial surgery. With the experience provided, endoscopic techniques can be safely implemented in surgery for BSCM. A combination of neuroendoscopic visualisation and neuronavigation might enable a targeted size of brainstem corticotomy. Endoscopy can currently be considered a valuable additive tool to facilitate the preparation and resection of BSCM.

**Supplementary Information:**

The online version contains supplementary material available at 10.1007/s10143-022-01793-5.

## Introduction

Intracranial cavernous malformations with their natural history and referring treatment modalities have been under investigation for a long time. However, there is still an ongoing debate on the best treatment modality for such lesions in highly vulnerable locations [3]. Brainstem cavernous malformations (BSCM) represent 9–35% of all intracerebral cavernous malformations [1]. Due to re-bleeding rates of up to 34.7%, once bleeding occurs, BSCMs frequently come along with progressive, devastating neurological deficits [15, 43].

Treatment strategies for BSCM vary significantly between neurosurgical departments worldwide, mainly depending on the surgeon’s individual experience. The indication and timing of surgery are still under debate. A large multi-step Delphi consensus on decision-making in the treatment of BSCM has been published recently to improve the quality of evidence [12]. In case of surgery, gross total resection is considered the gold standard of therapy in this delicate area [5, 36]. Therefore, various approaches have been described [32, 49, 51]. Microsurgical techniques were applied in most of them. Meanwhile, neuroendoscopic techniques have proven to be a beneficial add-on in posterior fossa surgery [6, 20, 29].

However, since the protection of adjacent structures, the definition of convenient entry points to the brainstem and the assurance of gross total resection are critical factors for surgical success, neuroendoscopy may also contribute to favourable outcomes in this challenging pathology. This study aims to investigate the potential benefits of neuroendoscopic techniques in different approaches to BSCM.

## Methods

### General aspects and study population

A retrospective analysis of a prospectively maintained database from 01/2010 to 01/2020 was performed. The presented study population is a consecutive series of all patients who underwent BSCM resection within the authors’ department. All procedures have been carried out with the same technique by the senior surgeon (JO) with experience in endoscopic techniques for 15 years at the start of this series. Data acquisition and processing were approved by the local ethics committee of Saarland, Germany. Data assessment consisted of medical documentation, perioperative radiographic imaging, intraoperative video documentation and follow-up examinations. Video documentation included separate high-definition records of the microsurgical and the endoscopic part, respectively.

### Surgery

Indication for surgery was set if the patient showed red flag symptoms (progressive neurological deficits, deterioration of consciousness or cardiovascular dysregulation due to brainstem compression) or if symptomatic re-bleeding was comprehensible. Risk stratification was performed using the Lawton-Garcia grading scale for BSCM [18]. A detailed, individual, three-dimensional preoperative planning was performed based on MRI, DTI and fibre tracking data.

The central part of the surgery was performed microscopically (Pentero, CarlZeiss GmbH, Jena, Germany). Additional endoscopic techniques were applied manually at different time points of the procedure. Manoeuvring of the optics was free-handed. Endoscopic 2D visualisation was used only. In most cases, endoscopy was applied for visualisation purposes. In particular situations, preparation, coagulation or resection purely under endoscopic guidance was performed (supplemental video). Endoscopic equipment was accessible at all times during surgery. It included a set of various rigid-rod lens Hopkins optics, as well as a high-definition visualisation and recording unit (AIDA, Karl Storz Endoskopie, Tuttlingen, Germany). Intraoperative computed tomography (Siemens Healthcare GmbH, Erlangen, Germany) was available for MRI/CT-based neuronavigation with StealthAir System (Medtronic, Minneapolis, MN, USA). At the time of deployment, the entire technical equipment was officially licensed for neurosurgical procedures in human patients.

Surgeries covered the following approaches: Suboccipital midline (*n* = 14), retromastoidal-supracerebellar-infratentorial (*n* = 4), binostril-transsphenoidal-transclival [30] (*n* = 1) and right-frontal-transventricular (*n* = 1).

### Data analysis

Medical data sets were evaluated regarding the preoperative clinical status and medical condition after surgery and follow-up. Due to significant variations in follow-up durations, medical condition and radiographic findings after 12 months were set as primary outcome parameters. Physical and mental health questionnaire SF-12v2 (Hogrefe Verlag GmbH & Co. KG, Göttingen, Germany) was used for standardised final health surveillance as available. Radiographic imaging was analysed for defining the exact localisation within the brainstem compartments preoperatively. After 6 and 12 months, postoperative MRI controls were reviewed independently in a blinded fashion. The extent of resection was evaluated 6 and 12 months after BSCM resection.

The intraoperative video material was analysed in detail regarding visualisation, endoscope-related morbidity, definition and volumetry of the BSCM compared to the size of the entry zone, illumination of the resection cavity and identification of residual cavernoma or bleeding spots. A synoptic video of endoscope-assisted BSCM resection was cut using Magix Software GmbH, Berlin, Germany.

The size of corticotomy was determined by a case-based analysis of the intraoperative video documentation related to the diameter of the implemented suction device (P.J. Dahlhausen & Co. GmbH, Köln, Germany). The suction device with a diameter of 3 mm was set as reference, and the incision size was measured in relation using GNU Image Manipulation Program (GIMP V.2.10.30). Volumetric analysis of the BSCM was performed using preoperative MRI imaging in axial, coronal and sagittal projections (SECTRA PACS, Sectra Medical Systems GmbH, Köln, Germany). All measurements were schematically illustrated using 3D-graphic software (Tinkercard, Autodesk GmbH, München, Germany).

## Results

### General

A total of 20 procedures for BSCM have been performed in 19 patients (8 female, 11male) between January 2010 and January 2020. Complete data sets were available for all patients. The mean age at the surgery date was 53.5 (± 11.1) years. The average volume of the BSCM was 5.4 (± 5) cm^3^. An associated DVA could be identified and preserved in 4 cases (20%). Multiple cavernous malformations in the context of familiar disposition were seen in 2 patients (10%). Acute bleeding of the BSCM was seen in 16 cases (80%) before surgery. Four cases were admitted to the authors’ department with progressive clinical deterioration but without signs of acute bleeding in preoperative MRI studies. The average BSCM classification after the Lawton-Garcia grading system was grade III (grade I–grade V). There were no high-risk classifications > grade V. All but one case underwent surgery within an acute (0–3 weeks; *n* = 12) or subacute (3–8 weeks; *n* = 3) timespan after BSCM haemorrhage. One patient experienced BSCM bleeding > 8 weeks before surgical resection. The mean operation time was 126.6 (61–209) min. A detailed characterisation of the individual cases is listed in Table 1.

**Table 1:** General information on the study population and clinical outcome (*f*, female; *m*, male; *CN*, cranial nerve; *H&B*, House and Brackman score; *CN*, cranial nerve; *FMD*, fine motor dysfunction)

Case	Sex	Age at surgery (years)	Location of BSCM	Acute bleeding	Presurgical status	Status at discharge (mRS)	Follow-up	Radiologic follow-up	Duration of follow-up (months)
1	f	13	Pons, left	Yes	Sopor, palsy of CN VI/VII (H&B: 4)	Improved (1)	Palsy of CN VII (H&B: 2)	No residual	89
2	m	60	Dorsal medulla oblongata, left	Yes	Palsy of CN VII, hemiparesis, FMD	Worsened (6)	Death due to severe pneumonia	-	-
3	m	70	Pons, left	Yes	Palsy of CN V/VI/VII (H&B: 4), hemihypaesthesia	Improved (1)	Residual palsy of CN VII (H&B: 2), residual hemihypaesthesia	No residual	27
4	m	42	Dorsal pons, cerebellar peduncle, 4th ventricle	Yes	Vertigo	Improved (0)	No deficit	No residual	12
5	f	35	Medial pons	Yes	Palsy of CN V/VII/VIII	Improved (1)	Residual palsy of CN VII (H&B: 4)	No residual	62
6	m	59	Dorsal medulla oblongata	Yes	Dysarthria, hemihypaesthesia	Improved (2)	Mild dysphagia	No residual	15
7	f	48	Central pons	Yes	Bilateral palsy of CN VI	Stable (2)	Residual bilateral palsy of CN VI	No residual	12
8	m	63	Dorsal mesencephalon, cerebellar peduncle	Yes	Vertigo, hemihypaesthesia	Worsened (3)	Residual palsy of CN IV and residual hemihypaesthesia	No residual	12
9	m	54	Central pons	Yes	Hemihypaesthesia, dysarthria, dysphagia	Stable (2)	Internuclear ophtalmoplegia	No residual	23
10	f	58	Dorsal pons	No	Severe headache, paraesthesia	Worsened (3)	Hemiparesis left (3/5), palsy of CN III left	No residual	25
11	m	58	Dorsal pons, cerebellar peduncle	Yes	Severe headache, FMD right hand, vertigo	Improved (1)	Residual mild FMD right hand	Marginal ischemia within cerebellar peduncle; no residual	39
12	m	64	Dorsal pons, cerebellar peduncle	No	Vertigo, severe headache	Improved (1)	No deficit	No residual	51
13	m	56	Dorsal pons	Yes	Hemihypaesthesia, dysphagia	Worsened (2)	Palsy of CN III	No residual	23
14	f	29	Ventral pons	Yes	Palsy of CN VI/VII, hemiparesis, FMD	Improved (0)	No deficit	No residual	12
15	m	46	Mesencephalon, right	No	Palsy of CN IV/V/VI/VII (H&B: 3)	Improved (1)	Residual palsy of CN IV	No residual	15
16	f	50	Upper pons, left	No	Palsy of CN V, hemiplegia, severe dysarthria	Improved (2)	Residual hemiparesis (4/5), slight dysarthria	No residual	54
17	f	62	Upper pons, right	Yes	Headache, hemiparaesthesia	Improved (0)	Second surgery after 6 months (case 20)	Residual cavernoma with re-bleeding after 6 months	13
18	m	69	Upper pons, right	Yes	Right hemiparesis, severe headache, dysarthria	Improved (2)	Residual mild hemiparesis (4/5)	No residual	16
19	f	73	Ventral mesencephalon	Yes	Diplopia, palsy of CN III, hemiparesis (3/5)	Stable (2)	Mild hemiparesis (4/5), residual palsy of CN III	No residual	16
20	f	62	Upper pons, right	Yes	Palsy of CN III and CN VII (H&B: 4)	Stable (1)	Residual palsy of CN III and CN VII (H&B: 2)	No residual	13

**Fig. 1:**
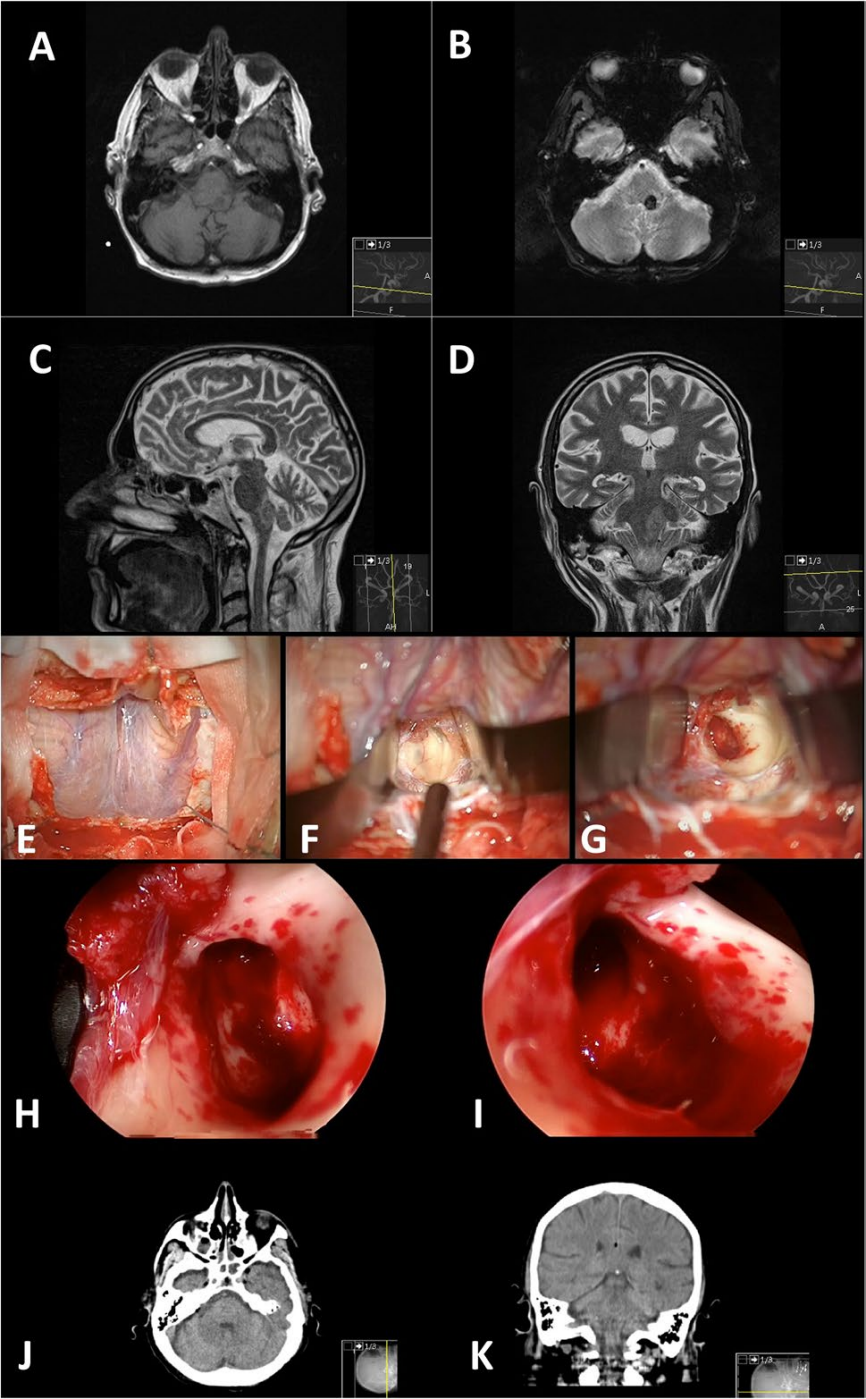
Illustrative case of a 59-year-old male patient with BSCM reaching the pial surface at the dorsal medulla oblongata left. Preoperative MRI studies are shown in **A**–**D**. The surgical approach was a suboccipital midline craniotomy with telovelar access to the brainstem (**E** + **F**). After microsurgical resection (**G**), the cavity is scrutinised with angled endoscopes (**H** + **I**). Note the slightly haemorrhagic spots in **H** + **I** most likely due to tearing the tissue — even with endoscopic techniques. The postoperative CT scan showed no complications (**J** + **K**)

**Fig. 2:**
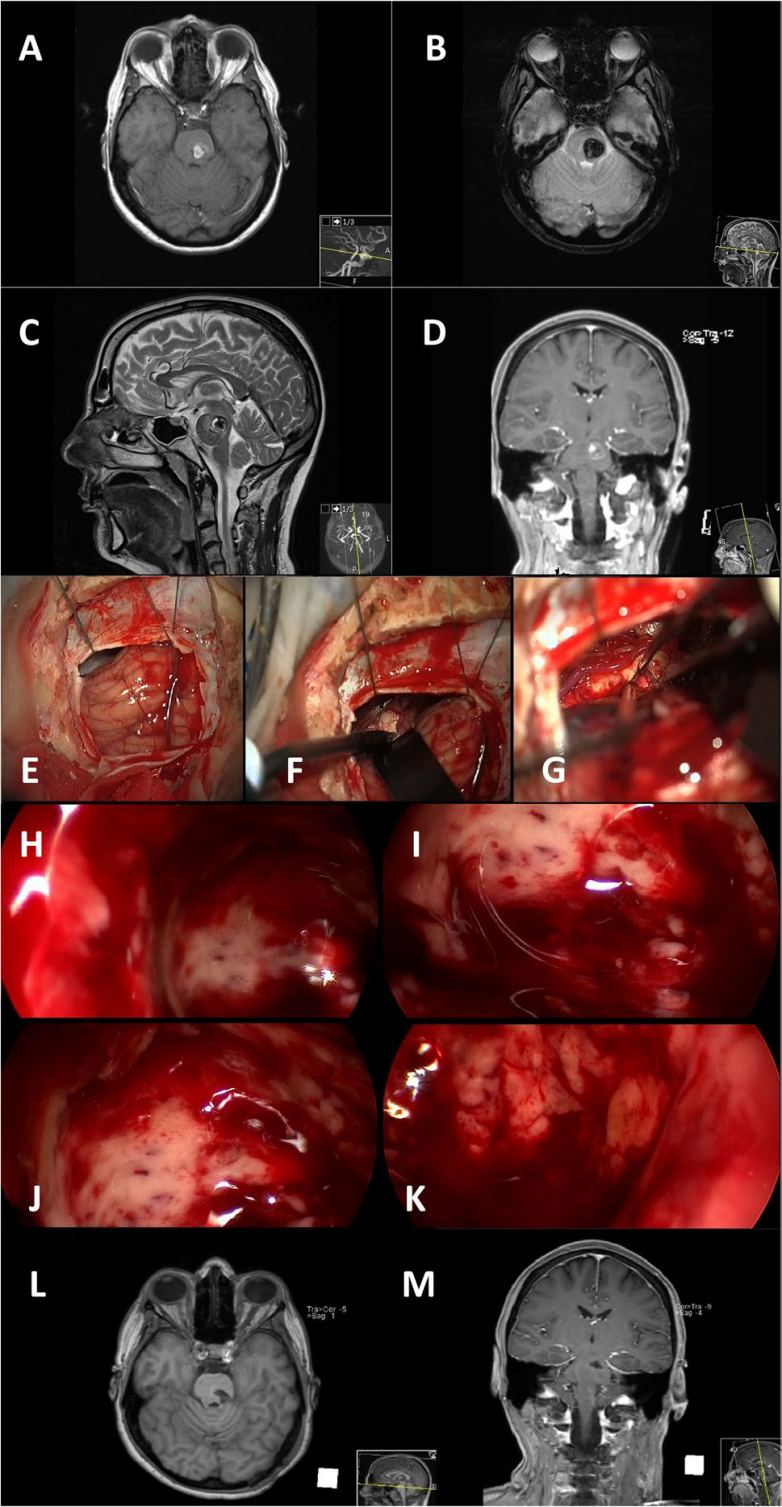
Illustrative case of a 46-year-old male patient suffering from BSCM located in the upper pons left. Preoperative MRI studies are shown in (**A**–**D**). A retromastoidal craniotomy is performed to access the lesion under gentle retraction of the cerebellum (**E** + **F**). After corticotomy, the BSCM is resected under microscopic view. The microscope’s limited visualisation of the cavity is shown in **G**. Endoscopic 360°-illumination of the resection cavity is shown in **H**–**K**. Postoperative MRI showed no residual cavernoma (**L** + **M**)

**Fig. 3:**
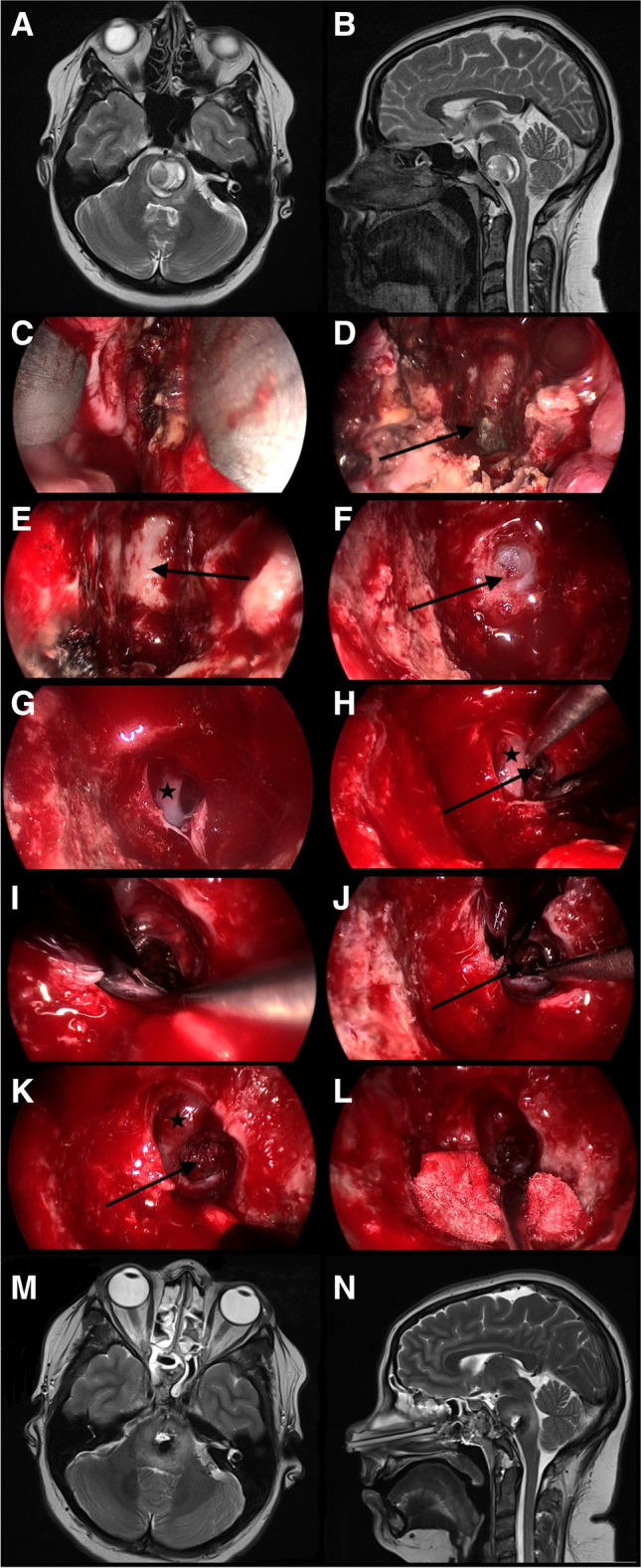
Illustrative case of a 29-year-old female Patient with BSCM reaching the ventral surface of the pons. Preoperative MRI studies are shown in **A** + **B**. Transsphenoidal, transclival, pure endoscopic resection was performed (**C**–**L**). The sphenoid sinus was inspected (**D**; arrow), and the clivus (**E**; arrow) was resected by drilling. After opening the dura mater (**F**; arrow), the basilar artery could be identified (**G**; star). Corticotomy (**H**; arrow) was performed laterally to the basilar artery (**H**; star), and the BSCM (**J**; arrow) was resected consecutively. Inspection of the resection cavity (**K**; arrow) showed no residual cavernoma or bleeding. Postoperative MRI studies showed significant pressure relief and gross total resection (**M** + **N**)

**Fig. 4:**
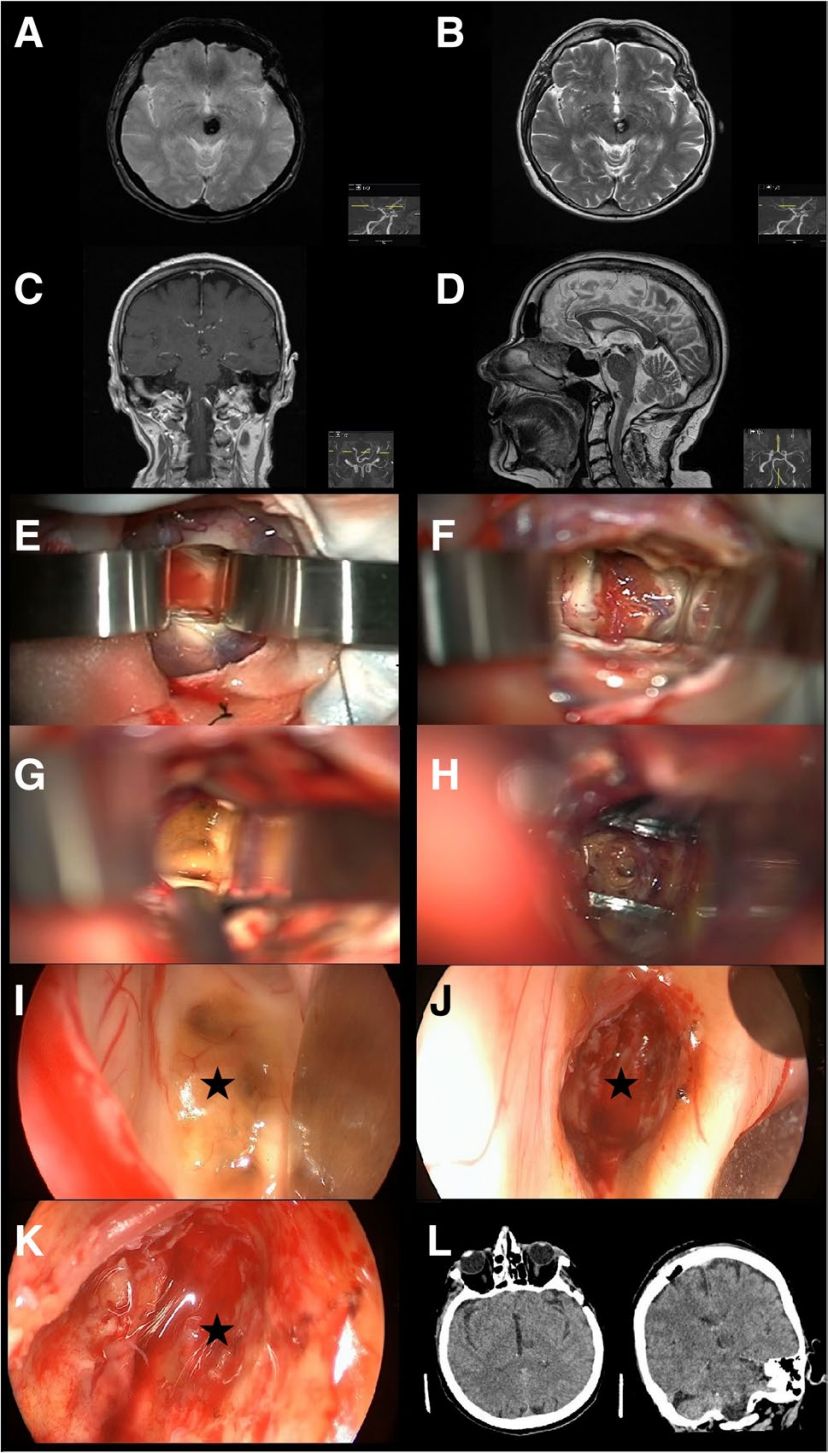
Illustrative case of a 73-year-old female patient suffering from BSCM located ventrally within the mesencephalon. Preoperative MRI studies are shown in **A**–**D**. To reach the entry point, a right frontal, transcortical approach to the lateral and third ventricle was performed to reach the entry point (**E**–**G**). The cavernoma was identified by endoscopic inspection (**I**; star) and resected afterwards (**H**). Final cavity examination with differently angled endoscopes revealed no remnant cavernoma tissue nor significant bleeding (**J**–**K**; star). The postoperative CT scan showed no infarction or bleeding (**L**)

A suboccipital midline approach (Fig. 1) was performed in 14 patients. Retromastoidal supracerebellar infratentorial approach (Fig. 2) was performed in 4 cases. Binostril transsphenoidal transclival approach (Fig. 3) and right frontal transventricular approach (Fig. 4) were performed in one case. All surgeries but two (transsphenoidal and transventricular approaches) were performed in a semi-sitting position.

### Surgery

Endoscopic techniques have been applied in all procedures. The binostril transsphenoidal transclival approach was performed purely endoscopically.

**Fig. 5:**
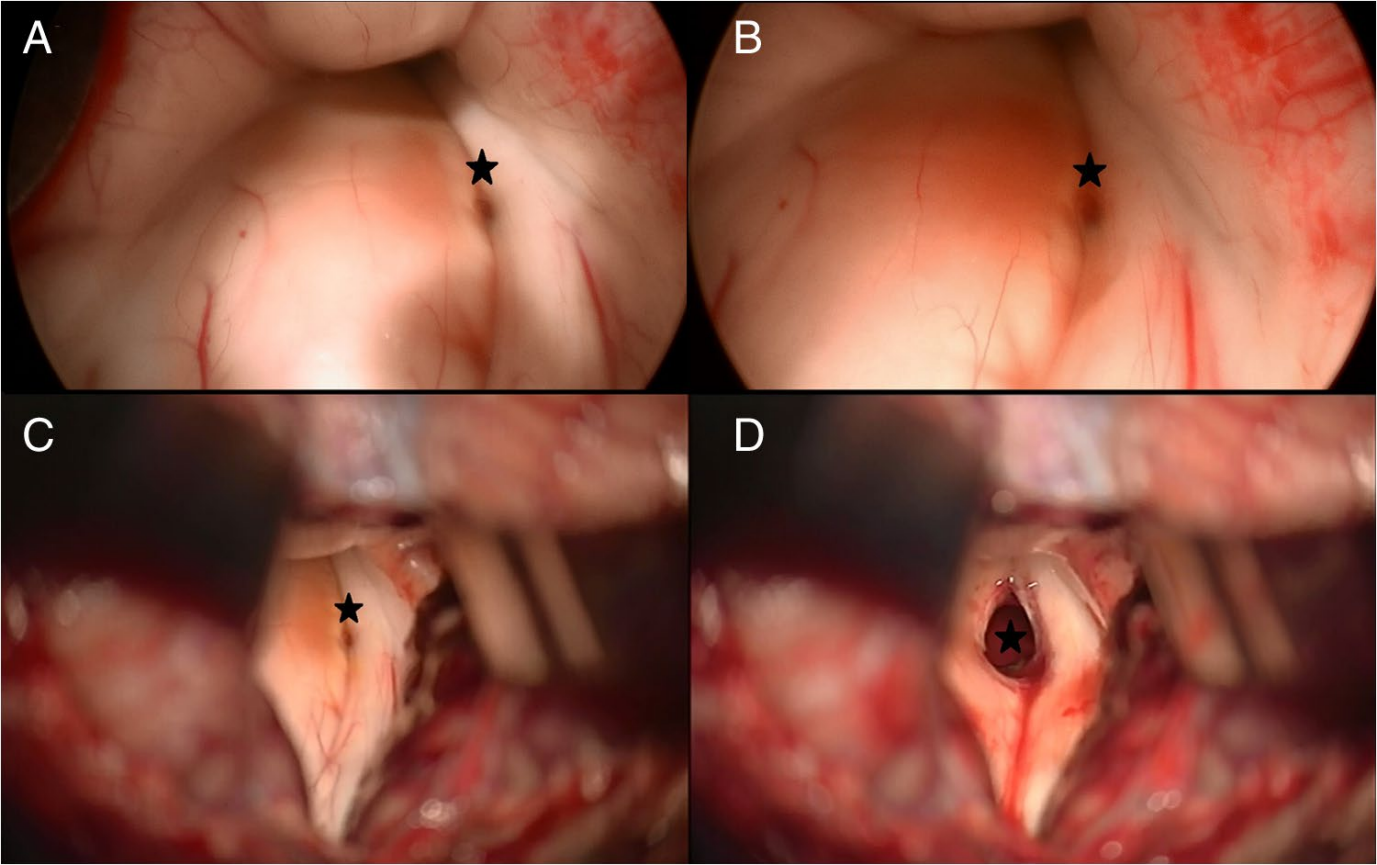
Definition of the entry point for corticotomy by the endoscope. The breakthrough of cavernoma tissue at the pial surface was inspected endoscopically (star, **A** + **B**). Minimal corticotomy was performed at the defined entry point (star, **C** + **D**)

In all cases, the favoured entry point into the brainstem was defined under free-handed, bimanual endoscopic guidance and neuronavigation (Fig. 5). By combining endoscopy and neuronavigation, the size of corticotomy could be limited to an average of 4.5 × 3.7 (± 1.0 × 1.1) mm. The median relation between the size of corticotomy and the maximum dimension of BSCM was 9.99% (1.2–31.39%). A scaled, schematic illustration of the BSCM location within the brainstem, compared to the size of the entry point, is shown for each case in Table 2.

**Table 2:**
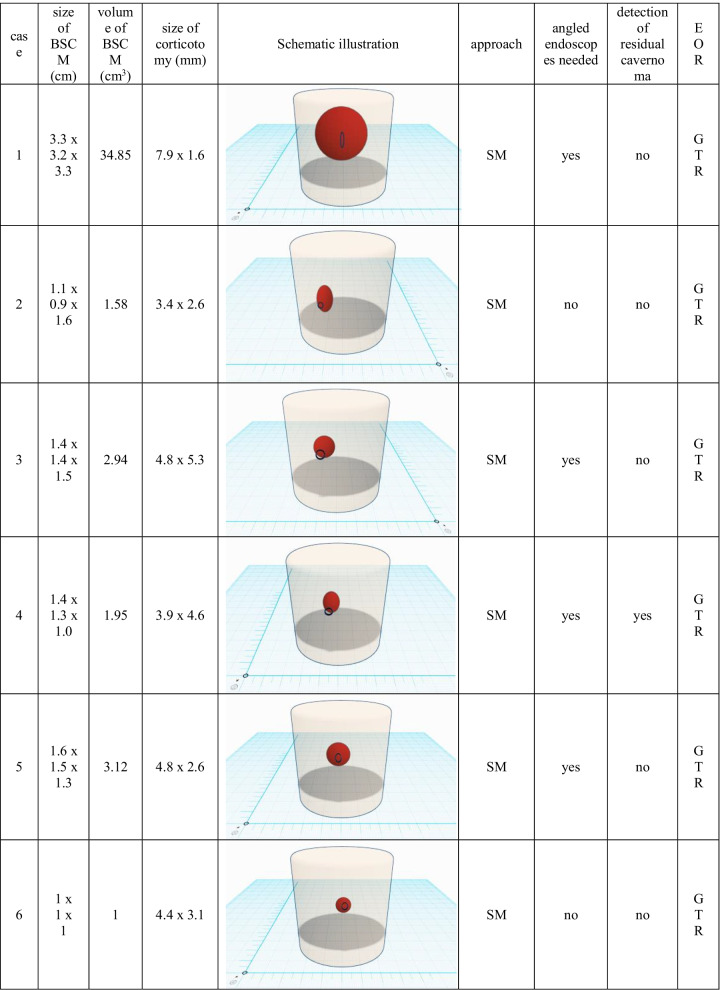

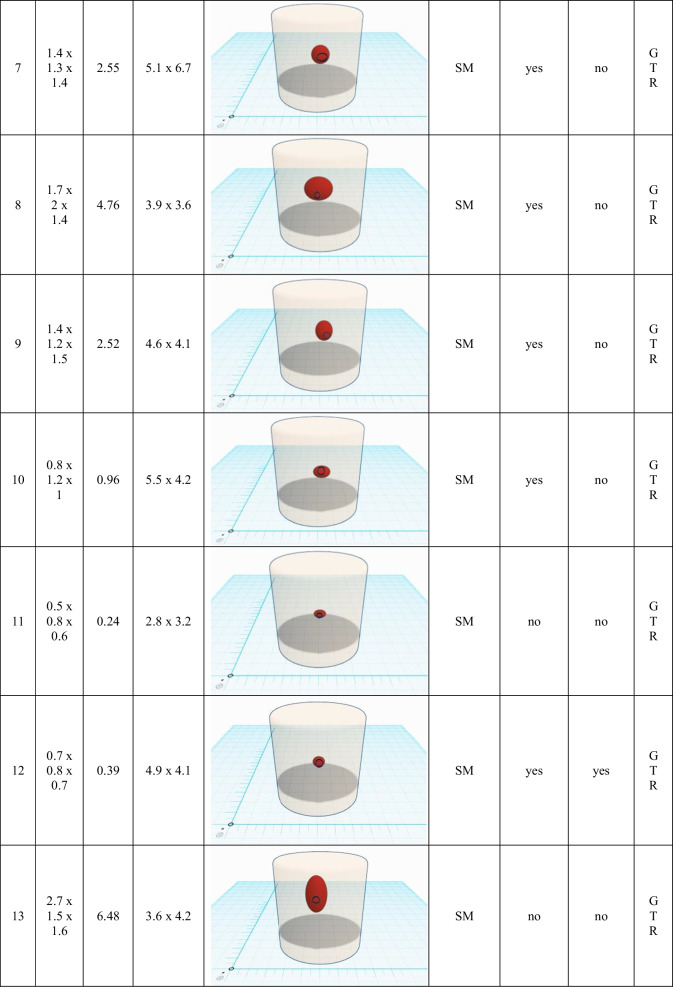

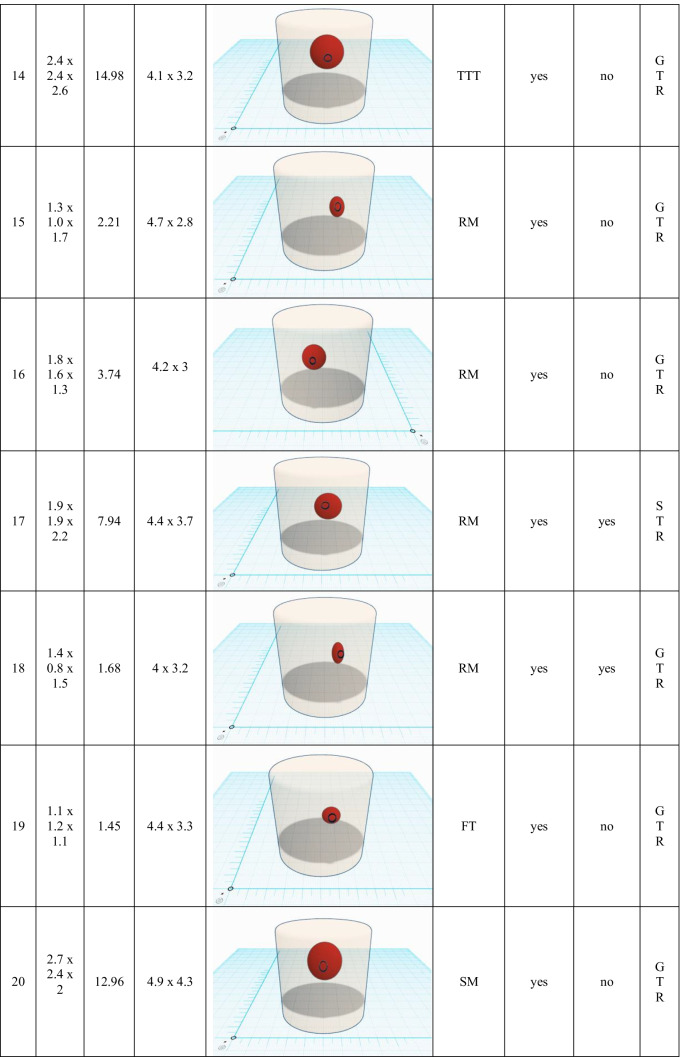
Endoscopy-related outcome and surgical aspects. A scaled schematic illustration of BSCM (sphere) location within the brainstem (basket) and volume compared to the size of corticotomy (circle) is shown for each case. (*cm*, centimetre; *mm*, millimetre; *SM*, suboccipital midline; *TTT*, transnasal-transsphenoidal-transclival; *RM*, retromastoidal; *FT*, frontal-transventricular; *EOR*, extend of resection; *GTR*, gross total resection; *STR*, subtotal resection)

Resection of the BSCM was performed under microscopic guidance with periodic endoscopic inspection in all but the transsphenoidal case. Through the miniature corticotomy, pure microscopic inspection of the entire resection cavity was feasible and considered sufficient in 4 cases (20%). In 16 cases (80%), the microscope alone could not inspect the resection cavity entirely. By applying endoscopic visualisation, extensive 360° illumination of the resection cavity was feasible in all cases. A 0° telescope with a range of up to 120° view was applied primarily. Angled telescopes (30°, 60°) were additionally needed in 16 (80%) cases.

All surgeries were finished under the assumption of gross total resection. There was no endoscopy-related contusion of surrounding brain tissue or eloquent structures. A detailed summary of the endoscopy-related surgical outcome is shown in Table 2.

### Outcome and follow-up

An improvement of clinical symptoms immediately after surgery was documented in 12 cases (60%). Four cases (20%) remained clinically stable. Four surgeries (60%) resulted in postoperative worsening compared to the preoperative status. Postoperative new cranial nerve palsies were seen in 4 cases (20%). One patient suffered from terminal liver insufficiency and died 10 days after surgery due to severe pneumonia unrelated to the BSCM surgery.

Postoperative imaging the day after surgery assured the absence of significant haemorrhage in all cases. One case (5%) showed marginal, local postoperative ischemia within the cerebellar peduncle but without clinical correlation. One patient (5%) showed residual cavernous malformation with re-bleeding 6 months after initial surgery (Table 1; case 17). This patient’s second surgery was performed, and gross total resection could be achieved (Table 1; case 20).

Mean follow-up was 27.8 (12–89) months. Clinical follow-up after 12 months was accessible in 18/19 (94.7%; one death) patients. None of those 18 patients showed clinical deterioration regarding initial postoperative symptoms. Eleven (61.1%) patients showed improved postoperative clinical status after 12 months. Seven patients (38.9%) remained in the postoperative clinical status after 12 months. Three patients (16.7%) were completely free of symptoms after 12 months.

Standardised health questionnaire results with SF-12 were available in 11/19 patients (57.9%) with a median time after surgery of 34 months. The study population showed a reduced mean physical health summary score of − 1.53 standard deviations (± 1.18) compared to the German norming sample from 1998. The mental health summary score was − 0.11 (± 0.99) and turned out to fit in between the average distribution compared to the norming sample.

## Discussion

### General considerations

BSCM is a rare condition. The scientific focus is on different treatment modalities, the ideal time point of surgery or radiotherapy and surgical approaches [16, 18, 34, 37, 43]. Indication for surgery is mainly given by a symptomatic lesion that is surgically accessible [47]. Al Mefty and Spetzler pointed out that the definition of “surgically accessible” can be interpreted widely and that it rather depends on the institutions’ experience in the treatment of BSCMs [5]. Accordingly, it seems crucial to optimise surgical precision and effectiveness to the highest level possible. Implementing neuroendoscopic techniques has improved surgical success in various posterior fossa pathologies, e.g. in intrameatal vestibular schwannoma resection [20, 24, 29, 31, 42]. Even comparably rare indications such as resection of optic pathway cavernous malformations have been treated successfully under endoscopic guidance [10, 46]. However, reports on endoscopic techniques in procedures for BSCM remain very limited to several case reports and small series (Table 3) [4, 14, 21, 22, 30, 33, 39, 40, 45].

**Table 3:** List of literature reports on endoscopic techniques in surgery for BSCM

Publication	Type of study	Patients	Endoscopy	Approach
Sandalcioglu et al., 2002 [30]	Retrospective, single-centre series	12	Partly endoscopic assisted	Variable
Sanborn et al., 2012 [29]	Case report	1	Fully endoscopic	Transnasal, transclival
Linsler & Oertel, 2015 [14]	Case report	1	Fully endoscopic	Transnasal, transclival
Nayak et al., 2015 [23]	Retrospective, single-centre series	4	Fully endoscopic	Transnasal, transclival; retrosigmoidal; supracerebellar
He et al., 2016 [24]	Case report	1	Fully endoscopic	Transnasal, transclival
Gomez-Amador et al., 2017 [27]	Case report	1	Fully endoscopic	Transnasal, transclival
Erickson et al., 2018 [28]	Case report	1	Fully endoscopic	Transnasal, transclival
Alikhani et al., 2019 [25]	Case report	1	Fully endoscopic	Transnasal, transclival

### Surgery

A minimally invasive approach and manipulation within the brainstem are crucial to preserving eloquent brain tissue and structures. The two-point method, published by Brown et al., aims towards limiting surgical corridors. In some cases of BSCM, when the direct approach crosses eloquent tissue, it even recommends an alternative, sometimes more demanding approach [8]. Moreover, extralesional and intralesional bleeding must be differed precisely to avoid unnecessary preparation [34]. The favourable entry point was defined under endoscopic view (Fig. 5). The subsequent corticotomy could be limited to an average of 4.5 × 3.7 (± 1.0 × 1.1) mm. The minimal invasiveness is strengthened by the median relation between the size of corticotomy and the maximum dimension of BSCM of 9.99% (1.2–31.39%) (figures within Table 2). Surgical invasiveness due to preparation on the brainstem surface could be limited effectively. Unfortunately, there is no systematic analysis of the size of corticotomy and its effect on the surgical success or clinical outcome in microsurgical procedures available. Ichinose et al. use “Microroll Retractors” to dilate the corticotomy for better visualisation, especially of deep-seated BSCM [23]. Striving for the same objective, angled endoscopes were applied in this study to enlarge the field of view without stressing the brainstem cortex. For deep-seated lesions with the need for surgery, in particular, endoscopic visualisation could enable a panoramic inspection without the need for enlarging the corticotomy. However, since endoscopically assisted resection of BSCM is the standard technique for BSCM resection in the authors’ department, no internal control group could be assessed. Accordingly, the presented results lack statistical proof of significance. Yet, the authors presume that a definition of convenient entry points and angled endoscopic inspection without enlarging the corticotomy contributes to a less invasive surgical preparation.

In this study, endoscopes were applied free-handed and manoeuvred manually at different time points of the procedure. Whilst the authors are used to inserting the optics purely under endoscopic visualisation, modern microscopes enable a synergistic combination of both techniques to improve orientation and safe handling. Such microscopic integration might advance getting familiar with endoscopic techniques in this specific indication. In the presented cases, only 2D visualisation was used. However, with an increasing frequency of endoscopically assisted or purely endoscopic procedures in neurosurgery, technological solutions for a stereoscopic view are demanded. In this context, 3D-exoscoscopes turned out to be somewhat applicable in spinal procedures [9]. Possible advantages of 3D-HD-endoscopic visualisation, as described for transsphenoidal pituitary surgery [44], remain elusive regarding BSCM resection. The authors used endoscopy mainly for additional inspection purposes. In limited cases, preparation, coagulation or resection is carried out under pure endoscopic guidance. Currently, the endoscope should be considered as an adjunctive tool for detailed inspection in addition to the microscope. Close-up visualisation might facilitate the identification of residual BSCM and bleeding spots within the resection cavity. Especially during preparation and resection of BSCM in deep cavities, angled endoscopes could effectively reduce the need for tractive enlargement of the corticotomy to inspect the entire cavity. However, the microscope remains the core visualisation tool so far. Yet, the implementation of advanced endoscopic visualisation technologies in the future may form a basis for future BSCM resection purely under 3D-endoscopic guidance.

The presented cases underline the possibility of a safe implementation of neuroendoscopy in various approaches to BSCMs. There were no intraoperative complications associated with the endoscope in this study. However, precautious manoeuvring is essential since the optics are inserted free-handed and guided manually. Manual handling, especially of angled endoscopes, underlies a certain learning curve [41]. Whilst surgical results of endoscopic transsphenoidal procedures could be shown to significantly improve after 20–50 cases [25, 28], it seems evident that such numbers can hardly be achieved for BSCM. Thus, endoscopically assisted resection of BSCM should be reserved for extensively trained neuroendoscopists. The senior surgeon (JO) already had broad expertise in cranial and spinal endoscopic techniques. This may lead to the absence of endoscopy-related complications within the presented series and highlights the necessity of neuroendoscopic experience in this specific pathology.

Achievement of gross total resection remains the fundamental surgical goal. The risk of fatal re-bleeding due to remnant cavernoma cannot be emphasised enough [7, 52]. Especially in deep-seated lesions, microscopic insight into the resection cavity can be very limited [40]. This dilemma aggravates by minimising the surgical entry point into the brainstem, as shown in this series. In such cases, endoscopy can be of high value. As shown, endoscopic 360° inspection of the resection cavity was possible in all cases, even through the smallest corticotomy of 2.8 × 3.2 mm. Due to the limited number of patients included and the absence of a statistical control group, a probabilistic analysis of detection rates with the endoscope cannot be provided. However, assurance of a gross total resection might be supported by additional endoscopic inspection and should be evaluated in further studies. Garcia et al. recently reported a recurrence rate of 6.6% in his large series of 213 patients with BSCM in over 20 years. Blind spots and misinterpretation of the resection cavity’s surface were considered significant contributors defining morbidity and cure [19]. We strongly believe that the endoscope adds essential information for the neurosurgeon at this point. Especially considering the proposed right-angle-method [19], angled endoscopes might facilitate detailed inspection of potential blind spots. However, one patient (5%) showed re-bleeding in the presented study due to recurrence 6 months after initial resection. Hence, the endoscopic visualisation should not be considered a guarantee for gross total resection. Undetected residual cavernoma tissue cannot be precluded despite the possibility of the resection cavity’s circumferential illumination. Yet, the endoscopic inspection might reduce the risk of unidentified remnant BSCM. Still, the study design, with its limited case numbers and the absence of a microsurgical control group, does not allow a statistically convincing conclusion in this context, and further prospective studies are needed.

### Clinical outcome

Favourable clinical outcome after surgery for BSCM is reported in a majority of all cases. An improved or stable medical condition can be found in 61–91% [17, 34, 35, 51]. In the presented study, 80% of the patients showed an improved, or at least stable, clinical status after surgery. Furthermore, 61.1% improved after another 12 months of follow-up. Hence, the presented results seem very representative compared to previous studies.

Wu et al. reported a statistical trend of cavernous malformations involving cerebellar peduncle towards unfavourable short- and long-term outcomes [50]. In this study, four patients showed BSCM reaching into the cerebellar peduncle. Only one patient showed clinical deterioration after surgery, whilst the others had an excellent clinical outcome after 12 months. Without strengthening it statistically, the presented results cannot support this thesis.

Though overall clinical outcome appears to be favourable in the vast majority, intraoperative morbidity should not be despised. With surgery-related morbidity of 20%, the presented study fits in between the reported morbidity rates of 10–37.3% [1, 2, 15, 38, 48]. However, standardised health questionnaires revealed below-average values of physical health compared to the German norming sample, whilst mental health scores were comparable to the average distribution within the norming sample. In the literature, the overall quality of life and mental health outcomes have been reported to be favourable after BSCM resection [11, 27]. Yet, a recent multimodal outcome analysis by Dammann et al. emphasised the complex interrelation between postoperative neurological deficits and impairment in quality of life. Cranial nerve deficits and brainstem symptoms, in particular, showed a significant impact on physical and mental quality of life even in favourable outcome patients [13]. Therefore, future studies on outcomes after BSCM resection should include detailed and standardised quality of life assessments to better represent individual outcomes [13, 26]. Compared to the available literature reports, implementation of endoscopic techniques for BSCM resection does not seem to increase surgery-related morbidity. Considering that the surgeon’s experience in neuroendoscopy is highly relevant in this context, an interindividual variety in morbidity rates must be assumed. However, even with the implementation of additional intraoperative techniques such as endoscopy, clinical outcomes are still unsatisfactory, and further effort must be put into improving postoperative quality of life in this delicate population.

## Limitations

The presented study has several significant limitations. Even though there is limited literature on endoscopic techniques in BSCM surgery, this study contains a limited number of patients. The retrospective character makes it susceptible to information and selection bias. Follow-up periods varied noticeably between the presented patients. Although almost all patients underwent clinical and radiological examination after 12 months, subsequent treatment in peripheral or distant hospitals impedes a consequent long-term follow-up in all patients. The major limitation is given by the absence of an internal control group for detailed statistical analysis. Since the endoscope-assisted resection is the standard procedure for BSCM surgery within the authors’ department, no internal data for such research was available. The presented conclusions must therefore be interpreted with restraint. This study aims to illuminate the potential benefits of implementing neuroendoscopy in BSCM surgery. Hopefully, other institutions will be inspired to share their experience, enabling an intensified scientific discourse allowing a robust statistical evaluation.

## Conclusion

With the experience provided, endoscopic techniques can be safely implemented in surgical resection of BSCM. A combination of neuroendoscopic visualisation and neuronavigation might enable a targeted size of brainstem corticotomy and an overall reduction of surgical invasiveness. Endoscopy can currently be considered an additive tool to facilitate the preparation and resection of BSCM. More extensive data is needed to enable statistical validation of these assumptions.

## Supplementary Information

Below is the link to the electronic supplementary material.

Supplementary file1 (MP4 203470 KB)

## Data Availability

All data acquired for this study is available for further inquiries.
